# Self-Amplifying RNA: Advantages and Challenges of a Versatile Platform for Vaccine Development

**DOI:** 10.3390/v17040566

**Published:** 2025-04-14

**Authors:** Thomas Vallet, Marco Vignuzzi

**Affiliations:** 1 A*STAR Infectious Diseases Labs (A*IDL), Agency for Science, Technology and Research (A*STAR), Singapore 138634, Singapore; thomas_vallet@idlabs.a-star.edu.sg; 2 Infectious Diseases Translational Research Programme, Department of Microbiology and Immunology, Yong Loo Lin School of Medicine, National University of Singapore, Singapore 118420, Singapore

**Keywords:** saRNA, mRNA, vaccine, alphavirus, limitations, safety, delivery, VEEV

## Abstract

Self-amplifying RNA is synthetic nucleic acid engineered to replicate within cells without generating viral particles. Derived from alphavirus genomes, saRNA retains the non-structural elements essential for replication while replacing the structural elements with an antigen of interest. By enabling efficient intracellular amplification, saRNA offers a promising alternative to conventional mRNA vaccines, enhancing antigen expression while requiring lower doses. However, this advantage comes with challenges. In this review, we highlight the key limitations of saRNA technology and explore potential strategies to overcome them. By identifying these challenges, we aim to provide insights that can guide the future design of saRNA-based therapeutics, extending their potential beyond vaccine applications.

## 1. Introduction

In recent decades, intervals between the most significant pandemics have been shortening. Events like the SARS outbreak in 2003, the H1N1 pandemic in 2009, the propagation of Zika in 2015, and the COVID-19 pandemic in 2019 occurred within shorter spans than historical patterns [1]. This trend, reflecting urbanization and environmental changes, underscores the urgent need for rapid and scalable therapeutic solutions, such as nucleic acid-based vaccines.

In the 1990s, DNA vaccines dominated the spotlight as the future of immunization. Pioneering work by Tang et al. (1993) demonstrated that DNA-coated gold particles could induce immunity, sparking excitement about their potential [2]. However, as DNA vaccines progressed to clinical trials, significant challenges became apparent, including poor immunogenicity and delivery issues. These failures led to widespread disillusionment, and nucleic acid vaccine research stagnated for many years. Initially dismissed due to its instability and susceptibility to degradation, mRNA gained little traction during this period, despite the foundational work of Wolf et al. (1990), which established protein expression from injected RNA in vivo [3]. The field continued to advance through years of dedicated research and clinical trials, leading to steady progress in mRNA technology. When COVID-19 emerged, these decades of innovation converged with an urgent global need, enabling the rapid development of the Pfizer–BioNTech and Moderna mRNA vaccines.

Key innovations—such as lipid nanoparticle (LNP) delivery systems and optimized RNA stability—enabled mRNA to emerge as a viable platform, shifting focus away from DNA-based vaccines [4]. The Pfizer–BioNTech and Moderna mRNA vaccines, developed in record time, not only mitigated the immediate threat of SARS-CoV-2 but also altered the vaccine ecosystem. These therapeutics’ ability to induce an immune response without the need for live attenuated pathogens associated with lengthy research and development makes them an attractive tool for the future. Moreover, the versatility of mRNA vaccines has opened the door to a broader range of applications against numerous pathogens and diseases. Unfortunately, despite the promise of these vaccines during an emergency, a notable challenge and limitation emerged during the pandemic: the rapid waning of immunity over time [5]. While initial protection against the SARS-CoV-2 variant is strong enough to prevent severe illness, hospitalization, and death, evidence has shown that mRNA vaccines’ effectiveness rapidly diminishes over months, making them no better than non-replicative, protein-based, subunit vaccines [6]. The gradual decline in antibody levels and the reduced effectiveness against new viral variants highlighted the need for innovative solutions.

Self-amplifying RNA (saRNA) represents a viable alternative to traditional mRNA vaccines. saRNA can be engineered by replacing the structural protein genes of the alphavirus genome with an antigen of interest while preserving the replication machinery intact to amplify the RNA transcripts. Frolov et al. (1996) demonstrated that such a structure enables the robust protein expression of an antigen that is strongly amplified by the replication machinery without forming infectious particles [7]. Following the re-emergence of mRNA as a safe and promising vaccine option, saRNA is currently regaining attention, with its first approval as a vaccine against SARS-CoV-2 in 2023 [8].

This review will examine key aspects of saRNA technology, focusing on its unique features, potential advantages over mRNA, and current challenges for gene therapy or vaccine applications. We will emphasize the core differences in its mechanisms of action and immune responses. We will then explore cutting-edge developments in saRNA replication machinery that can address stability, safety, or replication efficiency limitations. Finally, we will go through the patent landscape of saRNA to evaluate its potential for novel applications.

## 2. Background and History of Nucleic Acid Vaccines

### 2.1. The Principle of Genetic Immunization and DNA Vaccines

The basis of genetic immunization is the introduction of genetic material into host cells to induce an immune response. Unlike traditional vaccines, which rely on inactivated or attenuated pathogens, genetic vaccines provide the nucleic acid blueprint so that the host cells produce the specific antigenic proteins themselves. Subsequently, these antigens are presented at the cell surface, activating T cells and/or stimulating the production of neutralizing antibodies. In the early 1990s, DNA vaccines emerged as a promising method for eliciting both humoral and cellular immune responses. Tang et al.’s initial publication in 1992 proved that direct injection of DNA into mouse muscle could generate CD8+ MHC class I-restricted cytotoxic T lymphocytes [2]. This discovery sparked extensive research, with thousands of studies exploring DNA vaccines for various pathogens, including HIV, Hepatitis B, and influenza [9]. Despite encouraging preclinical results, DNA vaccines faced significant challenges. Indeed, clinical studies of first-generation DNA vaccines, primarily consisting of naked DNA, demonstrated that this platform induced only low levels of immunity, compounded with difficulties in efficient delivery to the nucleus of host cells and concerns over genomic integration [10].

### 2.2. mRNA Constructs: From Concept to Vaccine Application

In contrast to DNA vaccines, early studies on mRNA as a therapeutic tool were limited, as this platform was first introduced in the early 1990s [11]. These obstacles led to the decline in DNA vaccine research by the 2000s. In 1995, Pahl and Bauerle showed that synthetic mRNA encoding the influenza virus hemagglutinin (HA) protein could be translated into the HA protein in vivo, triggering a specific immune response in mice [12]. Over this decade, many studies focused on targeting cancer antigens rather than infectious diseases, with various cancer targets such as melanomas and lymphomas [13]. Cancer presented an opportunity for targeted and personalized treatment, allowing researchers to design unique markers specific to individual tumors. In contrast, infectious disease vaccines required systemic immunity and high antigen expression, which posed challenges given the unstable nature of early mRNA technology and the lack of robust delivery systems. The development of LNPs in the mid-2000s addressed this challenge by providing a more efficient and stable means of mRNA delivery [14].

As the understanding of mRNA technology progressed, several modifications were introduced to enhance its stability and minimize unwanted immune activation. One of the most important breakthroughs was the replacement of uridine with pseudouridine, a modified nucleotide that improves RNA stability and reduces immune recognition. Contrary to immune evasion via RIG-I, as initially hypothesized, the translation of pseudouridine-containing mRNA has been shown to be independent of RIG-I [15]. Modified versions like N1-methylpseudouridine have further become crucial in optimizing mRNA for therapeutic usage, notably enhancing translation [16].

In parallel, mRNA was optimized for improved stability and translation. At the 5′ end, an m7Gppp cap (cap-1) structure was developed, mimicking the natural cap found in host cell mRNA, protecting the transcript from exonuclease degradation, and facilitating recognition by the translation machinery. At the 3′ end, an extended poly(A) tail was added to enhance mRNA stability by prolonging its half-life in the cytoplasm, ensuring sustained protein production [17]. Finally, codon optimization was employed to improve the translation efficiency; by selecting synonymous codons that are more frequently used in the host’s translational machinery, researchers enhanced the production of the encoded protein, maximizing the vaccine’s effectiveness [18].

The development of mRNA vaccines encountered significant challenges before achieving clinical success, notably due to setbacks in DNA vaccine trials, particularly those targeting HIV-1. Indeed, the HVTN 505 trial designed to test a DNA prime-recombinant adenovirus type 5 (ad5) boost vaccine regimen failed to provide protection against HIV-1 in a group at an increased risk for the infection [19]. Moreover, the STEP trial, which used an ad5-based vector, failed to prevent HIV-1 infection in participants. This outcome mirrored results from analogous studies in simian immunodeficiency virus models with rhesus macaques, where the vaccine did not elicit the desired protective effects [20]. Facing such disappointments, the mRNA vaccine research was initially met with skepticism. Consequently, early clinical trials for mRNA vaccines were predominantly conducted in patients with advanced-stage cancers, such as stage IV malignancies, where no other treatment options were available [21]. While these early trials encountered such problems as immune overactivation and limited efficacy, they provided invaluable insights into optimizing delivery mechanisms and refining mRNA constructs.

Advances in mRNA technology eventually culminated in its use for infectious diseases, with the first major success occurring during the COVID-19 pandemic. Building on decades of research and optimization, mRNA vaccines encoding the SARS-CoV-2 spike protein were rapidly produced. These vaccines demonstrated remarkable efficacy in preventing severe illness and reducing virus transmission, with clinical trials showing efficacy rates exceeding 90% [22,23]. The rapid development of these vaccines underscored the adaptability of mRNA platforms, enabling swift responses to emerging pathogens. Furthermore, the ability to easily modify mRNA sequences allowed the development of updated vaccine-targeting variants, exemplifying the platform’s potential in addressing rapidly evolving infectious threats [24]. However, mRNA vaccine platforms present several limitations, such as stability and storage, low-level and short-lived expression, and efficiency of delivery.

Among these limitations, the waning of immunity induced by the mRNA vaccines over time is the most concerning. Despite protection against severe disease and symptomatic infection, immunity is diminished within months, even more so against emerging viral variants. Among these limitations, the waning of immunity induced by mRNA vaccines over time is concerning. Despite providing strong protection against severe disease and symptomatic infection, immunity diminishes within months, especially against emerging viral variants. This rapid decline necessitated a booster dose to restore protection levels in as little as three to four months [25]. This waning immunity can be attributed to numerous factors. While antibody titers induced by vaccination are naturally declining over time, mRNA vaccines tend to induce narrower and less durable T-cell responses compared to natural infections, therefore affecting the long-term efficacy of protection [26]. Moreover, the continuous evolution of SARS-CoV-2 associated with an accumulation of mutations in its spike protein enables viral variants to partially escape neutralization by pre-existing antibodies, as demonstrated in the case of the Omicron variant [27]. In comparison, traditional vaccines, such as viral vector-based vaccines, tend to elicit broader T-cell responses but generate lower peak antibody levels compared to mRNA vaccines [28]. Inactivated virus vaccines, like CoronaVac, induce broader antigenic exposure but overall lower immunogenicity. However, booster doses have been shown to restore both humoral and T-cell responses after waning immunity [29]. These observations raise critical questions about the durability of mRNA-induced immune responses and highlight the need for continuous innovations in vaccine design.

One promising alternative is the self-amplifying RNA platform, which builds upon the foundation of mRNA technology but incorporates unique features to address mRNA vaccine limitations.

## 3. Self-Amplifying RNA Innovation

### 3.1. Advantages of saRNA over mRNA Technology

saRNA vaccines offer many advantages over conventional mRNA vaccines, particularly in their ability to replicate within host cells, which enhances their immunogenicity while reducing the amount of starting material required. This self-amplification feature allows for the production of higher quantities of antigenic proteins from lower doses, making saRNA more efficient. For instance, Vogel et al. demonstrated that saRNA vaccines could achieve equivalent protection against influenza compared to conventional mRNA vaccines but at significantly lower doses. Specifically, whereas an mRNA vaccine required doses of 80 μg to elicit a protective immune response in mice, saRNA vaccines achieved a similar efficacy with 1.25 μg [30]. Similarly, a more recent study by McKay et al. highlighted the dose-sparing potential of saRNA vaccines in the context of SARS-CoV-2. In this study, an saRNA vaccine encoding a prefusion-stabilized spike protein elicited robust neutralizing antibody responses and protection in preclinical models at doses as low as 1 μg [31]. Moreover, Akahata et al. reported on Phase 1 clinical trials that their saRNA vaccine targeting SARS-CoV-2 induced comparable levels of neutralizing antibodies and T-cell responses as those seen in recipients of mRNA-based vaccines while utilizing significantly lower doses of antigen [32].

These findings underscore saRNA vaccines’ efficiency in achieving potent immunogenicity while minimizing resource requirements. This remarkable characteristic of saRNA is tied to the level and overall duration of antigen expression. A study utilizing liposome-protamine-RNA (LPR) nanoparticles to deliver a Venezuelan equine encephalitis virus (VEEV)-based replicon encoding the receptor-binding domain in mice showed that a subcutaneous injection of 2 μg of LPR-saRNA induced high and prolonged antigen expression in draining lymph nodes. The antigen expression levels were significantly higher than those of an LPR-mRNA vaccine and lasted at least two weeks. In contrast, expression from non-replicating mRNA persisted for only four days [33]. Similarly, a recent study in 2024 further demonstrated the extended antigen expression of the spike protein delivered through an saRNA platform with detectable levels in lymph nodes for up to 44 days post-vaccination, reinforcing the advantage of saRNA platforms in sustaining antigen presentation [34].

**Figure 1:**
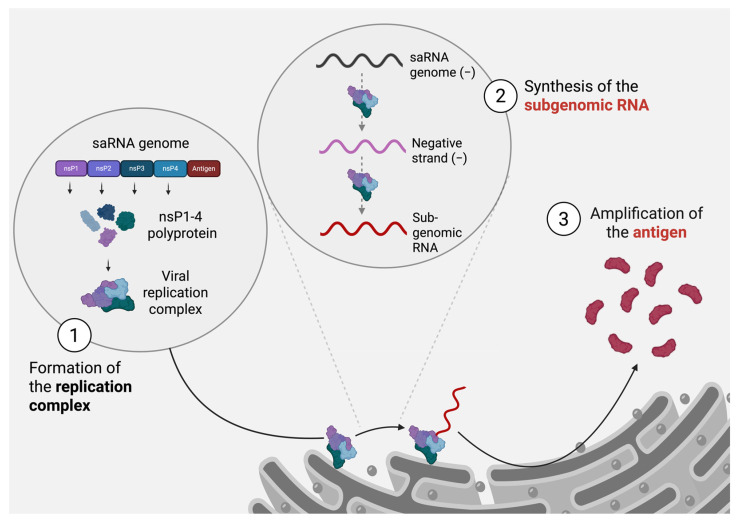
Main steps of the saRNA replication cycle within the host cell.

Most importantly, the efficiency of saRNA vaccines is the result of their underlying viral replication machinery. Replicons, self-replicating RNA molecules, are central to saRNA technology. Derived from different virus families, these structures retain the non-structural elements essential for viral replication while deleting some or all of the structural elements involved in the egress and production of infectious virus particles. A pioneering study by Kaplan and Racaniello on poliovirus replicons, published in 1988, provided a foundation for the development of replicons across virus families [35]. Later, replicons derived from the genome of the Hepatitis C virus (HCV) were invaluable in understanding HCV replication mechanisms and testing potential antiviral therapies [36]. Alphavirus replicons, developed in the same period, showcased the versatility of the replicon vectors in expressing heterologous genes. Mainly derived from the Semliki Forest virus (SFV) and the Sindbis virus (SINV), these replicons were engineered to replace structural genes with genes of interest, resulting in high levels of protein expression in host cells [37]. These early applications highlighted the potential of replicon systems as not only tools for studying viral replication but also for developing non-infectious vectors for gene expression and vaccine delivery. Building upon the foundation of replicon technology, saRNAs were developed using positive-strand RNA viruses as a prototype due to their highly efficient replication cycle and the ability of their nsPs to drive robust RNA amplification within the host cell cytoplasm (Figure 1).

Alphaviruses were first chosen as the backbone for saRNA due to their relative safety to work within research settings and the high amenability of their genome [38]. Alphaviruses possess an RNA genome organized into two open reading frames (ORFs). The first ORF encodes the non-structural proteins responsible for replicating the viral genome, while the second ORF, under the control of a subgenomic promoter and expressed as a subgenomic RNA, encodes the structural proteins. Upon viral entry into the cytoplasm, the nsPs are immediately translated from viral RNA to further form a replication complex on the membranes of the host cell, such as the endoplasmic reticulum or the plasma membrane. The nsP4 protein, encoding the RNA-dependent RNA polymerase, drives the viral genome replication. This process involves the synthesis of a complementary negative-sense RNA strand, which is then used as a template to produce an additional positive-sense RNA genome [39]. Meanwhile, subgenomic RNA is made during this stage, leading to the production of the structural proteins essential to the formation of virions. In saRNA vaccine design, the structural genes are replaced with an antigen-encoding sequence, allowing nsPs to drive robust antigen production within the cells.

### 3.2. Other Key Roles Played by Alphavirus nsPs That May Affect the Function of saRNA as a Therapeutic Vector

Throughout their replication cycle, alphaviruses employ sophisticated strategies to manipulate the host cell’s transcriptional machinery, prioritizing viral RNA synthesis over the host cellular processes. A key mechanism involves shutting off host transcription, which can be mediated through multiple avenues.

Old World alphaviruses, such as SINV and SFV, can interfere with host translation through capsid interactions with host ribosomes and the nsP2-mediated mechanism [40]. Through its proteolytic activity, nsP2 degrades essential components in the nucleus, such as the RNA polymerase II complex, including subunits required for transcription initiation. This degradation halts host transcription, reallocating cellular resources toward the production of viral components rather than antiviral factors [41]. As a result, the infected cell is essentially reprogrammed to focus efforts on expressing virally encoded proteins (or antigens expressed in their genomes). For New World alphaviruses, host shutoff is associated both with nsP2 activity and the presence of specialized RNA elements, such as a downstream loop within the 5′UTR [42]. These structures facilitate ribosome shunting, a process that allows the ribosome to bypass stable RNA secondary structures and initiate translation downstream, thereby ensuring continued viral protein synthesis under conditions of cellular stress. As a result, the cell is essentially reprogrammed to focus efforts on expressing virally encoded proteins (or antigens expressed in their genomes).

Dual enzymatic activities of nsP1, guanylyl-transferase and methyltransferase, were first identified by Ahola and Kaariainen in the mid-1990s [43]. These activities are crucial for the capping of viral RNA at the 5′ extremity, resulting in the formation of a cap-0 structure (m7G). This cap is essential for viral RNA stability and translation, as it protects the RNA from degradation by host exonucleases and allows for the efficient recognition by the eukaryotic translation machinery. The cap mimics host mRNA, facilitating evasion of specific immune sensors, such as Xrn1 [44]. In 2023, Law et al. uncovered another intriguing activity of nsP1: its ability to remove the cap-0 structure from host mRNAs. This decapping activity strips host mRNAs of their protective cap, exposing them to exonucleases like Xrn1, which can degrade the uncapped RNAs [45]. This targeted degradation of host mRNAs disrupts the cellular protein synthesis, creating a translational environment heavily favoring viral RNA. This mechanism enhances viral replication and contributes to the global shutoff of the host gene expression. Beyond its enzymatic roles, nsP1 is involved in the formation and localization of the viral replication complex. Acting as a membrane anchor, nsP1 interacts with host phospholipids, ensuring the replication machinery’s stable assembly on intracellular membranes, such as the endoplasmic reticulum or the plasma membrane [46]. This membrane association is essential for the spatial organization of replication and for protecting the viral RNA intermediates from host immune factors.

Lastly, nsP3 is an indispensable scaffolding component of the viral replication complex and an enzyme involved in various cellular interactions. Domains of this non-structural protein play different roles. The viral polymerase-associated one facilitates the interaction with other nsPs and the formation of the replication complex, while the V-like domain, containing a suppressor of RNA silencing activity, interferes with host antiviral defenses [47,48]. Known to interact with various host proteins, including those involved in stress granule dynamics, nsP3 also forms cytoplasmic RNA granules, which act as hubs for viral RNA synthesis and replication. These granules help compartmentalize the viral RNA and the replication machinery, protecting the viral genome from host defenses while concentrating the viral replication processes in one cell area [49]. The intricate functionalities of nsPs provide the foundation for saRNA’s unique capabilities as a vaccine, and it has already demonstrated efficacy in preclinical and clinical studies. However, limitations are associated with this emerging technology.

### 3.3. Limitations and Optimization Strategies

#### 3.3.1. Recombination Potential

As mentioned in Table 1, a primary concern when using alphavirus-based saRNA is the potential for recombination, especially in the presence of a co-infecting virus. Theoretically, within cells, the saRNA from the alphavirus replicon could recombine with the virus RNA, creating novel or altered viral strains. VEEV is a commonly used platform for saRNA design, as its high replication efficiency enhances antigen production compared to other alphaviruses [50]. The high replication rate of VEEV further increases the likelihood of mutations and recombination events, which could amplify the risk of chimeric virus formation. Hick et al. (2024) discussed the potential dangers of these recombination events, emphasizing that the self-amplifying nature of saRNA, especially when using high-replication viral platforms like VEEV as the backbone in saRNA design, could contribute to unintended genetic shifts, particularly in environments where viral co-infections or latent viral genomes are present [51]. This concern is not merely theoretical, as historical evidence of recombination between alphaviruses has demonstrated its implication in viral emergence. For instance, the Western equine encephalitis virus (WEEV) is a naturally occurring recombinant virus derived from SINV and the equine encephalitis viruses [52]. This recombination event resulted in a virus with unique pathogenic and ecological characteristics, highlighting the potential consequences of genetic mixing in alphaviruses.

One solution to this issue involves using non-replicating helper RNA systems, where essential replication sequences are deleted or modified. These modified helper RNAs are still transcribed and translated, but they lack the ability to replicate independently and are generally not packaged into viral particles. As a result, the likelihood of harmful recombination events with wild-type viruses in vivo is minimized compared to systems where the helper RNA is capable of replication [53]. However, the success of this method is not without challenges. The deletion of critical replication elements may impair the efficiency of protein expression or antigen production. Striking a balance between safety and functionality remains a significant hurdle for this approach. An alternative strategy is the trans-amplifying RNA (taRNA) system. This bipartite design separates the replicon into two distinct molecules: a helper RNA encoding the replication machinery and a separate RNA encoding the antigen of interest. While separating the transgene into different RNA molecules does not inherently reduce the likelihood of recombination with wild-type viruses, the use of a non-replicative helper RNA system may help minimize this risk. By ensuring that the helper RNA is non-replicative, the potential for recombination with wild-type viruses is reduced, as the replication machinery is not able to propagate independently. Beissert et al. demonstrated that this approach allows for a reduction in the antigen-coding replicon size without compromising immunogenicity, highlighting the potential advantages of the taRNA strategy for enhancing vaccine efficacy [54]. The use of high-fidelity RNA polymerases or ribozymes also represents an innovative way to minimize the chance of recombination. For example, the RNA polymerase ribozyme B6.61 has been isolated and exhibits superior extension and fidelity compared to traditional RNA polymerases [55]. These ribozymes can catalyze the synthesis of RNA with reduced error rates, which is crucial for maintaining the integrity of the replicon during amplification by minimizing the formation of defective viral genomes, which can contribute to recombination events [56].

These advances ensure that the RNA vector retains the intended sequence, decreasing the likelihood of unintended recombination with wild-type viruses during replication. Finally, innovative options include combining elements from different alphaviruses to create a modified replicon. A study by Wang et al. employed a heterologous backbone combining elements of VEEV and SINV, displaying robust immune responses against Chikungunya in mouse models and minimizing cytopathogenicity [57]. While chimeric saRNA constructs are not a primary focus, they could be an alternative in future applications in vaccine development. Finally, another strategy to reduce the risk of recombination is to introduce synonymous mutations throughout the saRNA genome at potential recombination hotspots. By modifying codons without altering the protein sequence, these mutations could reduce the homology between the saRNA and the RNA of circulating viruses. This strategy has been successfully applied in the context of polio live-attenuated vaccines, where synonymous mutations were introduced to reduce the chances of genetic reversion and recombination events [58]. Similar principles could be applied to saRNA vaccines to enhance their safety profiles.

#### 3.3.2. Cytopathogenicity

SINV and other alphaviruses induce cytotoxic effects due to their replication mechanisms, resulting in cellular damage and impaired host cell viability. Garmashova et al. proved the involvement of nsP2 in the cytotoxicity of the SINV replication cycle, an implication further elucidated by Treffers et al. through the identification of the phosphorylation pathway provoked by this non-structural protein [59,60]. In detail, nsP2’s activity involves interference with the JAK/STAT signaling pathway, crucial for antiviral responses, as well as the activation of mitogen-activated protein kinases like p38 and ERK1/2, contributing to stress granule disassembly and cellular apoptosis. nsP2 from VEEV has been extensively studied for its ability to inhibit host transcription through interactions with host nuclear components, leading to a shutdown of host gene expression and an eventual induction of apoptosis [61]. Furthermore, its high replication rate exacerbates cytotoxicity by overburdening the host cell’s machinery with deleterious effects on cell viability. These cytopathic outcomes pose significant challenges to the safety and efficacy of saRNA vaccines utilizing SINV and VEEV backbones and underline the necessity of optimizing the non-structural regions of these viral platforms to minimize adverse effects while maintaining the replication efficiency and antigen production. Moreover, cytopathogenicity can reduce the time during which cells are presenting the antigen. Essentially, these viruses and their replicons destroy the cells in which they replicate—and this damage is difficult to assess in vivo, where saRNA may accumulate in certain tissues or reach unwanted compartments.

The introduction of point mutations within the replicon backbone is one way to minimize its cytotoxicity. Lundstrom et al. evaluated the introduction of synonymous mutations into the nsP4 of an SFV replicon to reduce the cytotoxicity and temperature sensitivity. The modified saRNA displayed improved safety profiles without compromising its ability to induce immune responses [62]. More recently, Gong et al. investigated the effects of adaptive mutations in the nsP region by subjecting VEEV saRNA to exogenous interferon pressure. Among the mutations appearing through this selective pressure, A4130C and C4924A, located in nsP3, were associated with a decreased replication rate but reduced cytotoxicity compared to wild-type saRNA. Q48P and I113F mutations located in the macrodomain of nsP3 diminished the innate immune response and reduced the translation inhibition and cell apoptosis [63]. Alternatively, deleting parts of the non-structural protein sequences involved in the host cell transcription shutdown or apoptosis is under investigation. As a proof of concept, Cherkashchenko et al. showed that the truncation of the nsP2 C-terminal domain in SINV reduced its ability to suppress host transcription without impacting the replication ability [64]. Varjak et al. explored the truncation of the nsP3 C-terminal domain in SFV. Interestingly, they demonstrated that this modification attenuated viral-induced cell damage [65]. Lastly, modifications to replication kinetics offer another avenue for optimization. Adjusting the strength of the subgenomic promoter has been shown to regulate the balance between replication and translation. Geiss et al. demonstrated that reducing the subgenomic promoter strength of SINV-based replicons alleviated the cytotoxic effects while preserving the antigen expression [66]. Conversely, Kim et al. proved that modifications in the subgenomic promoter of VEEV replicons significantly enhanced subgenomic RNA synthesis and protein expression without showing evidence of increased cytotoxicity in the tested cell lines [67]. nsP4 is an alternative target for improving the replication kinetics. Lemm et al. identified mutants within the polymerase domain of SINV’s nsP4 that can reduce the excessive replication rate [68]. This highlights that the nsP4 activity is a critical determinant of alphavirus replication efficiency. Indeed, modifications to nsP4 can influence the replication rate, making it a promising target for regulating replication kinetics in alphavirus-based systems [69].

#### 3.3.3. Immunogenicity

saRNAs have been observed to skew the immune response toward cellular immunity, resulting in enhanced CD8+ T-cell responses [70]. This phenomenon is attributed to the self-amplifying nature of saRNA, which leads to a stronger activation of antigen-presenting cells (APCs) and a more efficient presentation of antigenic peptides on major histocompatibility complex class I molecules crucial for the activation of CD8+ T-cells. The innate immune sensors within APCs recognize pathogen-associated molecular patterns present in saRNA, triggering signaling pathways that produce type I interferons and other pro-inflammatory cytokines essential to the activation and maturation of APCs [71].

As useful as this is for targeting intracellular pathogens or tumors, it could lead to a less robust antibody response compared to traditional protein-based or viral vector vaccines. However, the balance of the immune response can also depend on the design of the vaccine, including the use of specific adjuvants or modifications to optimize B-cell activation alongside T-cell responses. Indeed, a study on a dual-antigen saRNA vaccine demonstrated that it induced high neutralizing antibody titers against multiple SARS-CoV-2 variants comparable to those in patients. The dual-antigen strategy involves encoding two distinct viral antigens within the saRNA constructs, enhancing the immune response by targeting multiple proteins such as the spike and nucleocapsid proteins [70].

#### 3.3.4. Stability

Among the key design improvements derived from advances in standard mRNA technologies, meaningful progress has been made to enhance the efficiency and stability of saRNA constructs. A notable innovation is the development of CleanCAP reagents for efficient co-transcriptional capping during in vitro transcription. CleanCAP AU has emerged as an advantageous tool for producing saRNA. By mimicking the canonical 5’UTR starter of alphaviruses, CleanCAP AU provides a Cap-1 structure to the mRNA, which improves the translation and replication efficiency [72].

While this modification addresses a critical challenge in saRNA design, the use of certain modified nucleotides, such as pseudouridine, presents unique obstacles. Widely adopted in traditional mRNA vaccines to reduce immunogenicity, its application in saRNA is limited due to the potential loss of these modifications along replication. However, studies demonstrated that 5-methylcytidine, 5-methyluridine, and 5-hydroxymethylcytidine can still provide measurable benefits, suggesting that the bulk of the immunogenic response may occur early, during the initial stages of infection, before replication amplifies unmodified RNA [73].

### 3.4. saRNA Vaccines

#### 3.4.1. Preclinical and Clinical Trials

One of the most notable alphavirus-based saRNA vaccine trials was conducted during the SARS-CoV-2 pandemic with ARCT-154. This VEEV-based saRNA vaccine was modified to enhance both the replication of the construct and the expression of the SARS-CoV-2 antigen. This vaccine underwent clinical trials across several countries, including a large Phase 3 trial in Vietnam [74]. This randomized, double-blind, controlled trial demonstrated that two doses of ARCT-154 had an efficacy of 56.6% against any symptomatic COVID-19 while providing 95.3% of protection against severe COVID-19. Additionally, adverse events were mainly mild to moderate from local reactions associated with headache and myalgia. Further research has expanded on the use of ARCT-154 as a booster dose for individuals previously immunized with mRNA vaccines. A Phase 3 non-inferiority trial compared ARCT-154 to the BNT16b2 mRNA vaccine and found that ARCT-154 not only elicited a robust immune response comparable to the mRNA vaccine against the original Wuhan-Hu-1 strain of SARS-CoV-2 but also induced a superior immune response against the Omicron BA.4/5 variant [8]. This makes ARCT-154 an appealing option for addressing emerging variants of SARS-CoV-2.

In addition to ARCT-154, another VEEV-based saRNA vaccine, ARCT-021, has also shown promising results in clinical trials. A previous study on ARCT-021 demonstrated that the vaccine was well tolerated up to doses of 5 ug against variants of SARS-CoV-2 [75]. Building on these findings, the combination of ARCT-021 and ARCT-024 is currently under investigation in a Phase 1/2 clinical trial. This approach may provide an advantage in generating a more robust immune response, thus improving protection against variants that can exhibit immune evasion properties. These observations align with the findings from the GRT-R9109 clinical trials, another saRNA delivering the entire spike protein of SARS-CoV-2. Notably, clinical data showed that neutralizing antibodies persisted for at least 6 months following a booster dose, indicating durable protection [76].

In contrast, the COVAC1 study on the LNP-nCoVsaRNA vaccine provides a different perspective. This vaccine, delivered through LNPs, was tested in a Phase I/II trial with the primary goal of assessing its safety and T-cell immune response. The study found that the LNP-nCoVsaRNA vaccine was well tolerated, with mild-to-moderate adverse events, including local reactions. Despite these encouraging safety findings, the clinical trials reported lower-than-expected seroconversion rates, which raised concerns about the overall antibody response elicited by this platform [77]. However, subsequent clinical trials have shown that while antibody responses may be modest, the LNP-nCoVsaRNA vaccine can still induce significant T-cell responses, which are crucial for long-term protection, particularly in immunocompromised individuals [78]. This underscores the need for further optimization to enhance both humoral and cellular immunity to bring the LNP-nCoVsaRNA platform closer in line with other successful saRNA vaccines, like ARCT-154 and GRT-R9109.

Another significant contribution to the saRNA vaccine efforts toward SARS-CoV-2 comes from Gennova Biopharmaceuticals, which developed HGC019, the first saRNA vaccine from India designed to be thermostable. HGC019 first demonstrated promising preclinical immunogenicity. Building on these findings, saRNA vaccines were further evaluated through the development of GEMCOVAC-OM, designed to target the Omicron BA.1 variant of SARS-CoV-2. In Phase 2 and 3 clinical trials, GEMCOVAC-OM elicited higher anti-spike IgG titers and superior seroconversion rates compared to the ChAdOx1 nCoV-19, with a favorable safety profile. Notably, no severe adverse events were recorded during these clinical trials [79]. Furthermore, recent studies have shown that GEMCOVAC-OM induced a broader and more robust cellular immune response compared to adenoviral-vectored vaccines. In a Phase 3 randomized trial, boosting with GEMCOVAC-OM resulted in significantly higher frequencies of Omicron-specific B and T cells. These responses were not only directed against the original Omicron BA.1 variant but also extended to newer variants, including XBB.1.5 and BA.2.86. This broad immune activation indicates that GEMCOVAC-OM primes the immune system to recognize and respond to a wider range of SARS-CoV-2, making it a promising candidate [80].

Beyond SARS-CoV-2, saRNA has also been explored for other diseases, including influenza and rabies (Table 2). A recent study evaluated an saRNA construct encoding an H3N2 influenza antigen in preclinical models. Vaccinated mice exhibited high titers of hemagglutination inhibition antibodies, with responses persisting for several weeks post-immunization [81]. The saRNA vaccine also induced robust T-cell responses, including both CD4+ and CD8+ T-cell activation. In parallel, Pfizer has developed a series of saRNA constructs targeting multiple influenza strains, with some candidates progressing to Phase 1 clinical trials.

As for rabies, Stokes et al. first demonstrated that an saRNA vaccine encoding the G glycoprotein of rabies induced robust immune responses in animal models [82]. The findings suggest that the saRNA vaccine was detected at the injection site and in draining lymph nodes shortly after administration. Building on this, the clinical trial for RBI-4000, an saRNA carrying the G protein of rabies, was conducted to evaluate the safety and immunogenicity in humans. In this study, RBI-4000 was administered at varying doses on a prime-boost schedule, demonstrating de novo protective immunity in a significant portion of the participants; immunity was observed in 94% of the participants receiving 1 μg and 100% of those receiving 10 μg doses. No serious adverse events were observed, confirming the safety and efficiency of the vaccine for rabies prevention [83].

#### 3.4.2. Regulatory Obstacles

Despite the promising results seen in preclinical and early clinical trials, saRNA vaccines face significant regulatory hurdles that must be overcome to ensure their widespread adoption and commercialization. These challenges are particularly evident in areas such as manufacturing, standardization, and approval processes.

- Manufacturing challenges

Regulatory agencies, including the FDA and EMA, require robust and reproducible manufacturing processes to ensure the safety and efficacy of vaccines. For saRNA vaccines, scaling up production in cell-based systems must yield sufficient high-quality saRNA while preserving the integrity of the product and minimizing the contamination risks. However, current cell-based manufacturing systems are prone to batch-to-batch variability, which can impact both consistency and purity. Similar challenges have been observed in other vector-based platforms, such as adenoviral vectors, where variability in transfection efficiency and expression levels can affect the final product quality [84].

Moreover, while LNPs are still considered the most efficient delivery methods of mRNA-based vaccines, variability in the formulation process presents significant challenges. Inconsistencies in encapsulation efficiency and heterogeneous particle size can hinder large-scale production by affecting the stability, biodistribution, and immunogenicity of the construct [85].

Finally, the establishment of standardized guidelines should be a priority for saRNA vaccine production. Ensuring consistent manufacturing processes and product characterization is essential for both safety and efficacy. Additionally, a deeper regulatory understanding of the self-amplification mechanism will be critical to establishing reproducible quality control measures.

- Approval process

The regulatory approval process for saRNA therapeutics is still under development. While mRNA vaccines have received emergency use authorization during the pandemic of COVID-19, saRNA vaccines require distinct regulatory pathways due to their self-amplifying nature. Another key regulatory challenge is the lack of a clear framework for evaluating the specific risks of saRNA, including the potential over-activation of innate immune responses or unwanted cytotoxic effects. Data from the ongoing clinical trials will be crucial in addressing these concerns and refining regulatory approaches. Long-term surveillance for adverse events will also be essential to maintain patient safety.

## 4. Discussion

Advantages of saRNA across vaccine platforms

In recent years, the development of saRNA vaccines has experienced rapid progress, particularly following the COVID-19 pandemic. The urgency of developing effective vaccines led to a significant surge in patents and publications on saRNA platforms, with several major pharmaceutical companies exploring their potential. According to Google Scholar patent databases, the number of patents related to saRNA has increased drastically since 2020, reflecting growing interest in harnessing its advantages for infectious disease prevention. Numerous studies have demonstrated the potential of saRNA platforms to generate both strong immune responses and long-lasting protection, with promising results across various viral targets, such as the Zika virus and yellow fever viruses [86].

Compared to traditional mRNA vaccines, saRNA offers distinct advantages, including self-amplifying activity, which allows for robust and sustained antigen production. The amplification of RNA within the cell enhances the immune response’s magnitude and duration at much lower doses, reducing the need for high antigen loads and repeated immunizations.

Unlike viral vector vaccines that use a harmless virus, such as adenovirus, to deliver genetic material encoding the target antigen into cells, saRNA avoids the challenge of pre-existing immunity to viral vectors, which can affect efficacy [87]. Additionally, viral vector production can be complex and costly, whereas saRNA vaccines offer a simplified and potentially more cost-effective production process. Furthermore, while protein subunit vaccines, like the Novavax COVID-19 vaccine, contain purified pieces of the target pathogen, they generally require adjuvants to enhance the immune responses and may need multiple doses for long-term protection [88]. saRNA vaccines, on their side, induce both humoral and cellular immune responses due to the in vivo expression of the antigen. This dual immune response suggests that saRNA vaccines may provide superior protection, particularly against variants evolving to escape antibody recognition.

Finally, the self-amplifying nature of saRNA allows for lower doses of RNA, which reduces the potential for cytotoxicity and improves the safety profiles. The nsP in saRNA’s backbone can indeed be fine-tuned during vaccine design to further enhance safety, making saRNA an attractive alternative to other vaccine platforms that may present challenges in terms of safety and production scalability.

Optimization methods of saRNA constructs

Despite these essential improvements, traditional methods for optimizing the saRNA vaccine platform have often been tedious and time-consuming. They require labor-intensive steps to identify the most effective mutations for improving the replicon backbone. Early-stage strategies for enhancing replicon efficiency and reducing cytotoxicity typically involve trial-and-error approaches or limited sequence-specific analyses. For instance, while some studies focused on the introduction of point mutations or truncations in non-structural proteins, such as nsP2 and nsP4, these methods did not always yield the necessary depth of understanding regarding how different mutations across the replicon influenced critical parameters like replication efficiency, antigen expression, and safety. Researchers have also considered introducing random mutations across replicons by random mutagenesis, a time-consuming and inefficient method, since it requires screening a large number of variants. Indeed, while random mutagenesis can generate diverse mutant libraries, the subsequent screening process to identify desired traits remains a significant bottleneck [89].

Machine learning models could offer a more refined and efficient approach to optimizing saRNA constructs. One of the crucial steps in saRNA optimization is codon usage, which influences the translation efficiency in host cells. Traditional methods often overlook complexities such as the influence of rare codons, ribosomal stalling, or translational pausing [90]. By leveraging machine learning models trained on large datasets of genetic sequences and translation efficiency metrics, researchers can identify patterns that more accurately predict translation success [91].

Moreover, artificial intelligence (AI) could also be used to predict and improve the stability of saRNA molecules. This prediction is usually a complex task, as it depends on multiple factors, such as RNA secondary structure, GC content, and sequence composition. AI can accelerate this process by analyzing vast datasets of RNA sequences and their associated stability profiles [92]. A notable example is RNAdegformer, a deep learning model that predicts mRNA degradation at nucleotide resolution. This tool outperforms previous methods in predicting the degradation properties for COVID-19 mRNA vaccines, with predictions correlating better with in vitro half-life measurements [93]. Furthermore, AI-driven approaches, like RhoFold+, can accurately predict the 3D structures of single-chain RNAs from their sequences, providing important insights into RNA stability and function [94]. This progress underscores AI’s potential to revolutionize RNA stability predictions, facilitating the development of more effective and stable saRNA vaccines.

Other saRNA platforms

While alphavirus-based replicons have been the main focus of saRNA vaccine development, recent studies have also explored the potential of replicons derived from other viral families. This could provide unique opportunities to optimize saRNA vaccine platforms by tapping into different replication mechanisms and cell tropisms. Flaviviruses, like alphaviruses, possess an RNA-dependent RNA polymerase and associated nsPs, allowing for their efficient replication in both mosquito and mammalian cells. While both virus families share this capability, the specific characteristics of each, including replication dynamics and safety profiles, may offer distinct advantages for tailored vaccine designs targeting specific hosts [95].

Flaviviruses, such as ZIKV, have evolved to replicate across a broader range of tissues compared to alphaviruses, including neuronal and endothelial cells [96]. This ability to target diverse tissue types could be strategically leveraged in vaccine designs aimed at influencing specific immune responses. As for their unique replication features, flaviviruses undergo a circularization of their genome by the association of the 5′ and 3′ termini. This process is essential to ensure an efficient replication and packaging of the genome [97]. Moreover, this mechanism contributes to the stability of the viral genome. This characteristic could be exploited in saRNA platforms, allowing for a more controlled and efficient amplification of the vaccine mRNA within target cells while avoiding its degradation. Despite their promising advantages, flavivirus-based saRNA systems are not without challenges. More preclinical and clinical studies are required to confirm their safety and efficiency.

Taken together, saRNA represents a transformative addition to the vaccine toolbox, offering strong promises for addressing future pandemics and advancing therapeutic innovation. By incorporating cutting-edge technologies, saRNA has the potential to overcome the limitations of traditional mRNA platforms. Continued optimization of replicon design and delivery methods will be key to realizing the full potential of saRNA in vaccine development and beyond.

## Figures and Tables

**Table 1:** Overview of the challenges and solutions associated with saRNA development.

Limitation	Risk	Optimization Strategy
Recombination Potential	Presence of co-infecting viruses leading to novel viral strains	Non-replicating helper RNA systems
		Trans-amplifying RNA (taRNA)
		High-fidelity RNA polymerases or ribozymes
Cytopathogenicity	Cellular damage due to viral replication	Point mutations in nsP4 and nsP3
		Truncation of nsP2 and nsP3 domains
		Optimization of subgenomic promoter strength
		Alteration of replication kinetics via nsP4 modifications
Immunogenicity	Skewing of immune response toward cellular immunity	Dual-antigen saRNA design
		Optimizing adjuvants or modifications to enhance B-cell activation alongside T-cell responses
Stability	Degradation and immunogenicity issues with modified nucleotides	Use CleanCAP AU for improved capping efficiency
		5-methylcytidine, 5-methyluridine, or 5-hydroxymethylcytidine instead of pseudouridine

**Table 2:** Current landscape of clinical trials involving saRNA vaccines against infectious diseases.

Vaccine	Targeted Virus	Backbone	Key Features	Stage of Development	Clinical Trials (ClinicalTrials.gov Identifier)
ARCT-154	SARS-CoV-2	VEEV	Modified VEEV replicon for enhanced replication and antigen expression	Clinical Trials (Phase 3)	NCT05012943
ARCT-165	SARS-CoV-2	VEEV	Combination of ARCT-154 and ARCT-021 vaccines	Clinical Trials (Phase 1/2)	NCT05037097
saRNA-LNP	SARS-CoV-2	VEEV	Lipid nanoparticles formulated saRNA vaccine	Clinical Trials (Phase 1)	NCT04776317
PF-07852351, PF-07836391, PF-07836394, PF-07836395, PF-07836396, PF-07867426	Influenza	VEEV	saRNA targeting various influenza strains	Clinical Trials (Phase 1)	NCT05227001
AAHI-SC2	SARS-CoV-2	VEEV	saRNA encoding the spike protein, delivered by a nanostructured lipid carrier	Clinical Trials (Phase 1)	NCT05370040
GRT-R910	SARS-CoV-2	NA ^1^	saRNA booster	Clinical Trials (Phase 1)	NCT05148962
HGC019	SARS-CoV-2	NA^1^	-	Clinical Trials (Phase 1/2)	CTRI/2020/11/028476
GEMCOVAC	SARS-CoV-2	VEEV	-	Clinical Trials (Phase 2/3)	CTRI/2022/10/046475
RBI-4000	Rabies	VEEV	srRNA encoding rabies glycoprotein, optimized for low-dose delivery	Clinical Trials (Phase 1)	NCT06048770

^1^ Information regarding the alphavirus backbones for GRT-R910 and HGC019 is unavailable (NA).

## Abbreviations

The following abbreviations are used in this manuscript:

APCs	Antigen-presenting cells
CHIKV	Chikungunya virus
LNPs	Lipid nanoparticles
LRP	Liposome-protamine-RNA
nsPs	Non-structural proteins
ORF	Open reading frame
saRNA	Self-amplifying RNA
SFV	Semliki Forest virus
SINV	Sindbis virus
taRNA	Trans-amplifying RNA
VEEV	Venezuelan equine encephalitis virus
WEEV	Western equine encephalitis virus
ZIKV	Zika virus
